# A Novel Grid Strategy for Correlating Focal Macular Anatomic Changes With Focal Changes in Choriocapillaris Perfusion

**DOI:** 10.1167/iovs.65.14.5

**Published:** 2024-12-03

**Authors:** Farhan E. Hiya, Yuxuan Cheng, Mengxi Shen, Jianqing Li, Alessandro Berni, Sandy Wenting Zhou, Gissel Herrera, Robert C. O'Brien, Giovanni Gregori, Ruikang K. Wang, Philip J. Rosenfeld

**Affiliations:** 1 Department of Ophthalmology, Bascom Palmer Eye Institute, University of Miami Miller School of Medicine, Miami, Florida, United States; 2 Department of Bioengineering, University of Washington, Seattle, Washington, United States; 3 Department of Ophthalmology, First Affiliated Hospital of Soochow University, Suzhou, Jiangsu, China; 4 Department of Ophthalmology, IRCCS San Raffaele Scientific Institute, Milan, Italy; 5 Department of Ophthalmology, Tan Tock Seng Hospital, National Health Group Eye Institute, Singapore; 6 Department of Ophthalmology, University of Washington, Seattle, Washington, United States

**Keywords:** swept-source optical coherence tomography angiography (SS-OCTA), choriocapillaris perfusion, drusen, age-related macular degeneration

## Abstract

**Purpose:**

To establish the repeatability of choriocapillaris flow deficit (CCFD) measurements within a macular grid and then demonstrate the use of this registered grid strategy to follow CCFD measurements over time.

**Methods:**

Swept-source optical coherence tomography angiography scans were acquired (nominal size of 6 × 6 mm). For each scan, masks of hyperreflective foci, calcified drusen, and persistent choroidal hypertransmission defects (hyperTDs) were generated. These masks were then used to exclude these prespecified regions when calculating the CCFD percentages (CCFD%). Scans were registered, and CCFD% measurements were performed within 3-mm and 5-mm fovea-centered circles and within a fovea-centered grid (one box: 74 × 74 pixels). The 95% minimal detectable changes (MDC_95_) for CCFD% were calculated for each of the regions. This longitudinal grid workflow was then used to study eyes before and after drusen resolved.

**Results:**

Ninety eyes of 63 patients were identified: 30 normal eyes, 30 eyes with intermediate age-related macular degeneration (iAMD), and 30 eyes with hyperTDs. The MDC_95_ for the normal, iAMD, and hyperTD eyes within the 3-mm and 5-mm circles ranged from 0.85% to 1.96%. The MDC_95_ for an individual grid's box ranged from 3.35% to 4.67%, and for the total grid area, the MDC_95_ ranged from 0.91% to 1.40%. When tested longitudinally before and after the resolution of drusen using grid strategy, no significant differences in the CCFD% were observed.

**Conclusions:**

A grid strategy was developed to investigate targeted longitudinal changes in CCFD% associated with changes in optical coherence tomography biomarkers, and this strategy was validated using eyes in which drusen resolved.

Age-related macular degeneration (AMD) is a late-onset complex genetic disease and the leading cause of irreversible vision loss among the elderly worldwide.^1^ The diagnostic features that characterize nonexudative AMD can be identified by using optical coherence tomography (OCT) and OCT angiography (OCTA) imaging, and these diagnostic OCT biomarkers can be used to determine disease severity and the risk of disease progression.^2^^–^^13^ These OCT biomarkers include the area and volume of typical soft drusen and calcified drusen (CaD); the presence of reticular pseudodrusen (RPD), also known as subretinal drusenoid deposits (SDDs); the area of hyperreflective foci (HRF) within the retina, known as intraretinal hyperreflective foci and hyperreflective foci along the retinal pigment epithelium (rpeHRF); the presence of basal laminar deposits (BLamDs); the thickness of the outer retina; and the onset and area of persistent choroidal hypertransmission defects (hyperTDs), which develop into typical geographic atrophy (GA) and are related to complete RPE and outer retinal atrophy.^3^^–^^22^ OCT and OCTA imaging strategies also can identify both nonexudative and exudative macular neovascularization (MNV) along with the extent of the exudative macular fluid.^23^^–^^26^ Finally, swept-source OCT (SS-OCT) and SS-OCT angiography (SS-OCTA) imaging strategies are useful for imaging and measuring the macular choroidal vasculature, choroidal thickness, and the choriocapillaris (CC) because of its deeper penetration depth, minimal sensitivity roll-off, and longer ranging distance.^27^^–^^34^

The CC is the terminal capillary monolayer of the choroidal circulation that lies beneath Bruch's membrane (BM) and provides essential nutritional support to the RPE and photoreceptors.^35^^,^^36^ The loss of CC perfusion has been implicated as a risk factor for the progression of AMD.^37^ For example, in nonexudative AMD, studies using OCTA imaging have associated the presence of drusen with increased CC flow deficits (CCFDs) while other studies have shown the growth of GA to be associated with increased CCFDs.^33^^,^^38^^–^^46^ However, decreased central macular CC perfusion has also been associated with normal aging.^27^^,^^30^^,^^31^

Longitudinal studies are needed to determine if the loss of CC perfusion is a cause or a consequence of AMD progression and to characterize the temporal relationships between focal changes in CC perfusion with focal anatomic changes in AMD.^12^^,^^21^^,^^33^^,^^39^^,^^40^^,^^44^^,^^46^ The characteristic anatomic features of AMD measurable using this longitudinal strategy with OCT and OCTA imaging include the onset and growth of typical soft drusen, CaD, SDDs, HRF, BLamDs, photoreceptor loss, hyperTDs, and MNV.^3^^,^^5^^–^^13^^,^^16^^,^^23^^,^^47^^–^^52^ In this current study, we describe a novel grid strategy that provides a reproducible method for studying and associating changes in anatomic biomarkers of AMD with changes in CC perfusion at the same location over time. First, we established the 95% minimal detectable changes (MDC_95_) for CCFD% measurements using a set of repeated scans. Then, we used eight previously published cases,^53^ in which drusen resolved without atrophy to demonstrate the usefulness of this novel grid strategy to colocalize anatomic changes with CCFD measurements. This grid strategy and the established MDC_95_ will be useful and serve as a foundation in future longitudinal studies analyzing anatomical changes, such as HRF, soft drusen, CaD, RPD/SDD, BLamDs, photoreceptor loss, and hyperTDs, with changes in choriocapillaris flow deficits as cases progress from intermediate AMD to late AMD.

## Methods

Patients with normal eyes and eyes with nonexudative AMD were enrolled in an ongoing prospective, observational, SS-OCT imaging study at the Bascom Palmer Eye Institute. The institutional review board of the University of Miami Miller School of Medicine approved the study, and all patients signed an informed consent for this prospective SS-OCT study. The study was performed in accordance with the tenets of the Declaration of Helsinki and complied with the Health Insurance Portability and Accountability Act of 1996.

### Reliability Assessment

#### Study Population and Imaging Protocol

The reliability assessment involved a retrospective review of subjects enrolled in the above SS-OCT study to identify 30 normal eyes (eyes without any OCT pathological findings); 30 eyes with intermediate AMD (iAMD). defined by the absence of any large hyperTDs and the presence of at least one large druse with a minimum diameter of 125 µm and/or HRF; and 30 AMD eyes with hyperTDs. Eyes were excluded if other pathologies were present such as high myopia (≥6.00 diopters and/or axial length ≥26 mm), diabetic retinopathy, retinal vein occlusion, central serous chorioretinopathy, or macular neovascularization. Patients were imaged between August 2018 and March 2022, and these eyes were consecutively enrolled. For all three groups, if both eyes from a certain patient fulfilled the requirements of any group, then both eyes were included in the corresponding group(s). All subjects were imaged with three sequential, same-day SS-OCTA scans with a 6-mm × 6-mm scan pattern centered on the fovea (PLEX Elite 9000; Carl Zeiss Meditec, Dublin, CA, USA). The SS-OCTA instrument used in this study has a central wavelength of 1050 nm and a scan rate of 100,000 A-scans/s. Theoretically, the 6-mm × 6-mm scan pattern consisted of 500 A-scans per B-scan and 500 B-scans with each B-scan repeated twice, resulting in a uniform 12-µm spacing between A-scans and B-scans. Of note, the actual scan area and spacing vary among different eyes due to differences in axial length. This instrument has a full width at half maximum axial resolution of ∼5 µm in tissue and an estimated transverse resolution of ∼20 µm at the retinal surface. The OCTA flow information was created with the instrument's complex optical microangiography algorithm, and the interscan time between repeated B-scans is 6.1 ms. Scans were also excluded if they had a signal strength less than 7 based on the instrument's output or if they presented significant motion artifacts or insufficient illumination.

#### Imaging Processing and Quantification of CCFDs

Scans were exported from the SS-OCTA instrument and processed using various algorithms as described below. Drusen volume and area were computed using a standalone executable version of the Advanced RPE Analysis v0.10 algorithm (which is now available on the PLEX Elite 9000 software version 2.1).^49^^,^^52^ Drusen area and volume were reported in square root (sqrt) area and cube root (cubrt) volume, respectively. For the purposes of this study, persistent choroidal hyperTDs were defined as lesions that appeared bright with a greatest linear dimension of at least 250 µm on the en face image of the sub-RPE slab and confirmed on corresponding B-scans. The sub-RPE slab was created by segmenting the SS-OCTA volumetric scan from 64 to 400 microns below BM.^6^^,^^11^ Using a previously published algorithm for the semiautomated identification and quantification of hyperTDs, we created binary hyperTD masks for each scan.^9^^,^^50^ Additional binary masks were created for regions of peripapillary atrophy as well as regions that prevented the measurement of underlying CCFDs such as HRF, CaD, and vitreous shadowing.^53^^,^^54^ These regions were identified by a method based on the analysis of both en face images and corresponding B-scans, as described by Laiginhas et al.^5^^,6^ and Liu et al.^7^ All applicable masks for a given scan were merged to form a “combined mask,” and only areas outside of the combined mask were then used to calculate CCFDs.

**Figure 1.**
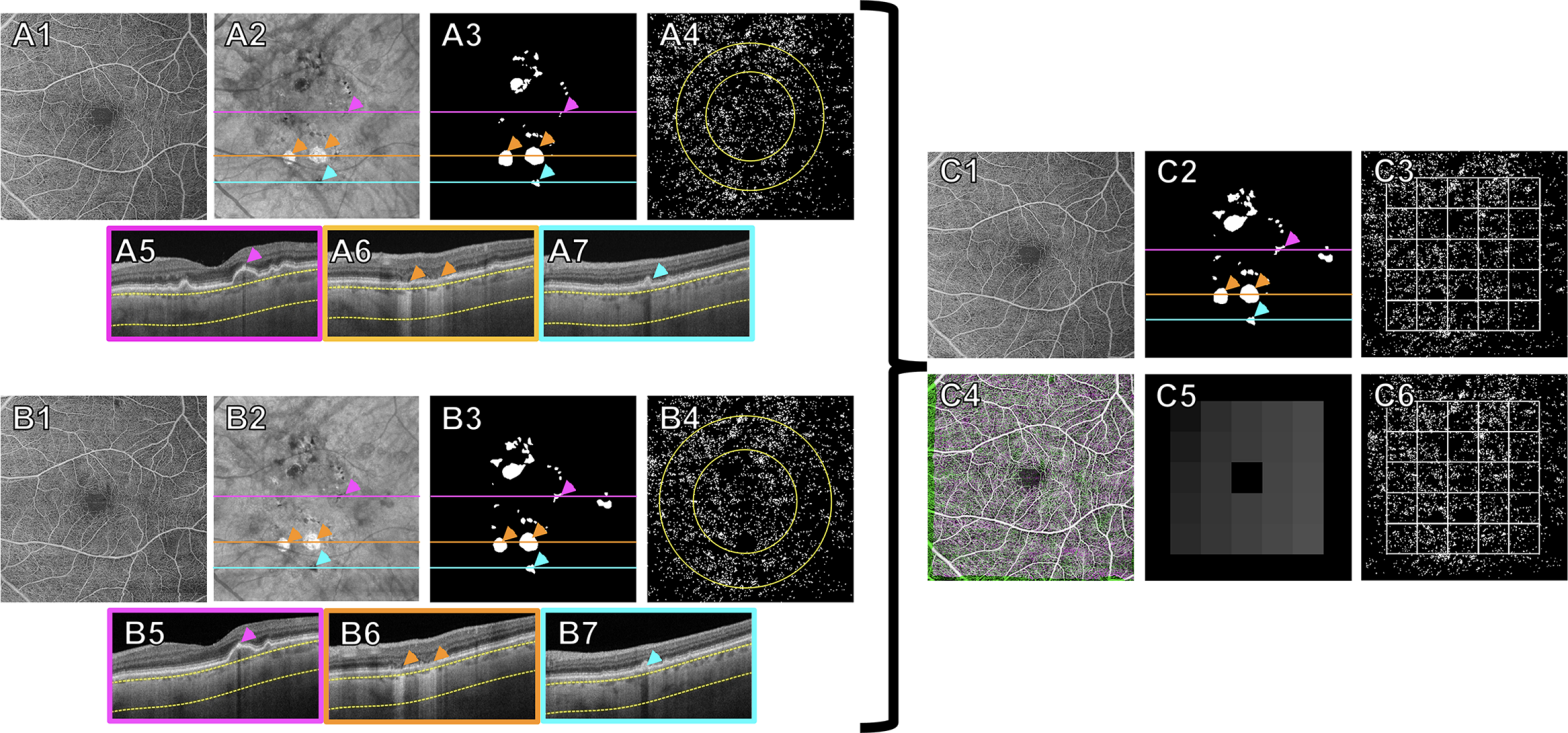
Workflow used to quantify changes in CCFDs between visits using a grid to divide the macula. (**A1**–**A7**) The baseline visit, (**B1**–**B7**) the 3-month follow-up visit, and (**C1**–**C6**) quantification of local, registered CCFDs. (**A1**, **B1**) The 6-mm × 6-mm retinal vascular OCT angiography maps used to perform registration between visits. (**A2**, **B2**) Sub-RPE en face images generated by a slab defined from 64 to 400 µm below the BM. (**A3**, **B3**) Combined binary masks generated by identifying and outlining HRF, hyperTDs, and CaD appearing in the scan. (**A4**, **B4**) CCFD binary maps excluding the regions defined by the combined masks shown in **A3** and **B3**, with 3-mm and 5-mm fovea-centered circles superimposed. *White areas* correspond to the CCFDs. (**A5**–**7**, **B5**–**7**) B-scans showing HRF (*purple arrowheads*), hyperTDs (*orange arrowheads*), and CaD (*blue arrowheads*), corresponding to the *purple*, *orange*, and *blue lines* marked in A2–3, B2–3, and C2, respectively. (**C1**, **C4**) Registration of en face retinal vasculature images of the baseline (**C1**) and follow-up (**C4**) scans. (**C2**) Final integrated mask, created by merging the registered combined mask from the follow-up visit (**B3**) with the combined mask of the baseline visit (**A3**). (**C5**) A grid (fovea-centered in this case) dividing the macula into 25 equivalent boxes. (**C3**, **C6**) Registered CCFD binary maps for the baseline (**C3**) and follow-up (**C6**) visits, excluding the regions defined by the integrated mask (**C2**), with the grid (**C5**) superimposed. The dimension of the grid is approximately 4.5 × 4.5 mm.

Figure 1 illustrates the process using the CCFD algorithm and the combined masks at each visit (Fig. 1A1–7, B1–7), followed by the registration of scans from different visits and the creation of a grid (Fig. 1C1–6) so that the same retinal region can be compared across visits. Each OCT volumetric scan, together with the corresponding combined mask and fovea location, was loaded into a custom CCFD algorithm for the quantification of CCFD percentages (CCFD%). First, the algorithm automatically segmented the CC as a 16-µm slab starting from 4 microns under BM. This slab was chosen to minimize the signal noise created by the RPE/BM complex while also considering the normal physiological location of the CC and the OCT's axial resolution.^34^ Segmentation lines were manually edited within the software if needed. Then, to account for signal attenuation due to abnormalities of the retina and/or RPE/BM complex such as increased RPE reflectivity or drusen, the algorithm inverted the en face CC structure image derived from the CC segmentation above and applied it to the en face CC flow image to compensate for signal intensity differences across the scan, thus reducing the rates of false-positive CCFDs due to OCT signal attenuation.^53^^,^^55^ The next step involved the removal of the retinal vessel projection artifacts and the areas identified in each scan's combined masks.^56^ Finally, the remaining scan regions were thresholded using the Fuzzy-C means global thresholding method, which has been shown to correlate well with the local Phansalker thresholding method when the Phanalker pixel radius is selected based on the intercapillary distance (ICD) of the CC in proportion to the OCT scan size (i.e., 2–4 pixels for 6 × 6 scans), while also proving to be robust across scans with varied OCT illumination levels.^28^^,^^34^ CC perfusion deficits less than 24 µm in the greatest linear dimension were excluded to remove FDs that likely corresponded to speckle noise and the physiological flow voids based on the estimated average ICD of the CC.^34^ The final outputs of the algorithm included a CCFD binary map with white dots representing flow deficits (e.g., Figs. 1A4, 1B4), as well as CCFD% measurements within the 3-mm and 5-mm fovea-centered circles, within the boxes of the grid, and for the entire grid. Of note, the actual scan area and actual area covered by the circles and the grid are not truly known and may vary among different eyes due to differences in axial length.^57^^,^^58^

#### Grid-Based Quantification of CCFDs

**Figure 2.**
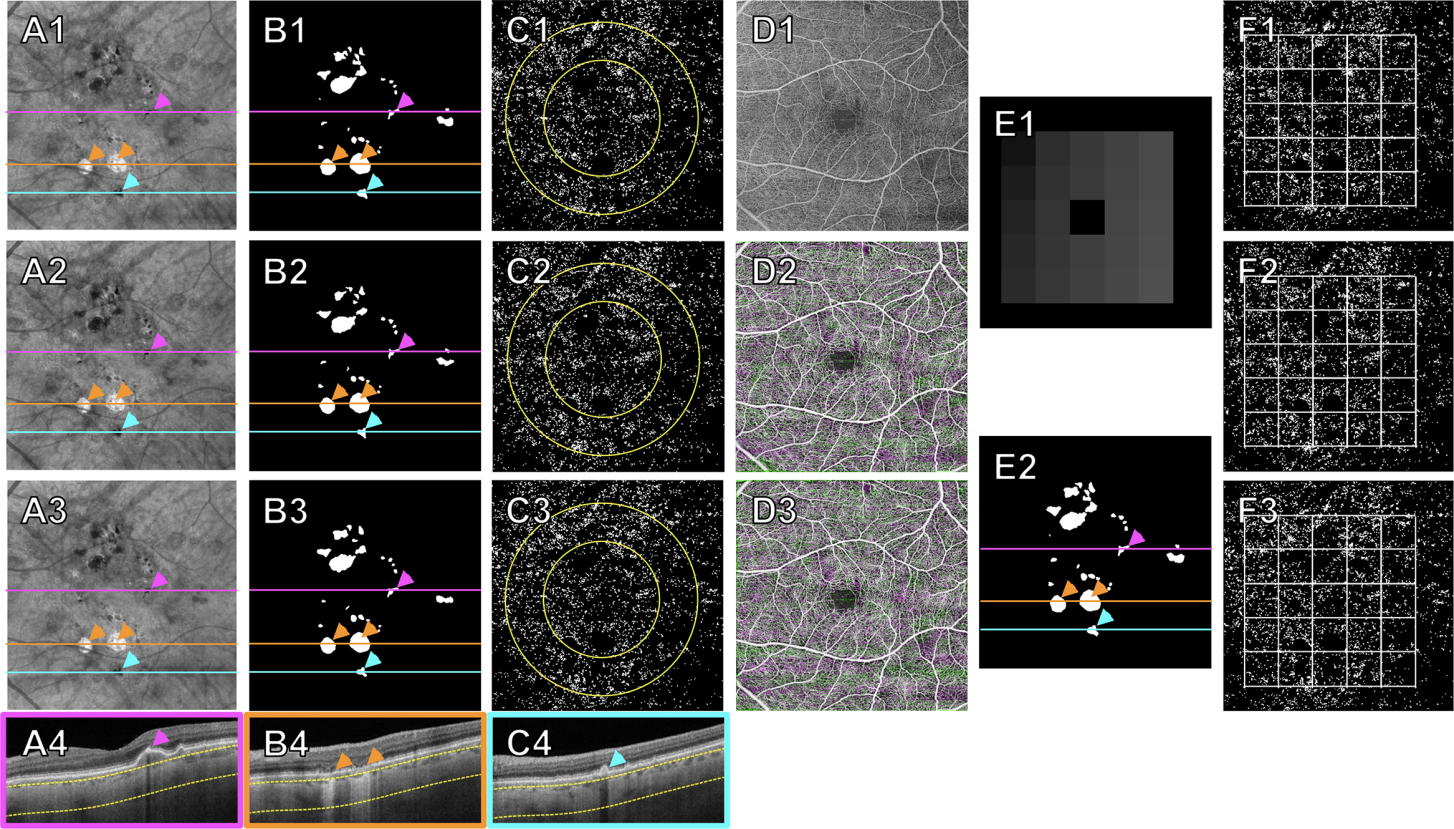
Workflow for registering three well-aligned repeated scans followed by the use of an integrated mask and a grid to quantitate CCFDs. In this example, the scan was found to contain persistent hyperTDs, HRF, and CaD. (**A1**–**3**) Sub-RPE en face images of the eye's three sequential scans, which were used together with corresponding B-scans to identify and outline the HRF, hyperTDs, and CaD. (**B1**–**3**) Resulting combined masks of HRF, hyperTDs, and CaD. (**C1**–**3**) Resulting CCFD binary maps, with 3-mm and 5-mm fovea-centered circles, after excluding the regions defined by their respective combined masks (**B1**–**3**). *White dots* correspond to the CCFDs. (**A4**, **B4**, **C4**) B-scans showing HRF (*purple arrowheads*), hyperTDs (*orange arrowheads*), and CaD (*blue arrowheads*), corresponding to locations marked by the *purple*, *orange*, and *blue lines* in **A1**–**3**, **B1**–**3**, and **E2**, respectively. (**D1**–**3**) Registration of en face retinal vasculature images of the three repeat scans. (**E1**) A fovea-centered grid dividing the macula into 25 equivalent boxes. (**E2**) The integrated mask of the case, created by merging all three registered combined masks (**B1**–3). (**F1**–**3**) Registered CCFD binary maps for each scan after applying this case's integrated mask and fovea-centered grid to each scan. *White dots* correspond to areas of CCFDs. The *white grid* highlights each individual box of the grid as well as the total grid area analyzed. The dimension of the grid is approximately 4.5 × 4.5 mm.

To determine the reliability of our measurements on repeated scans, we registered three sequential scans for each eye using a custom MATLAB (MathWorks, Natick, MA, USA) registration algorithm that registered the scans based on the en face images of the retinal vasculature, with the first scan serving as the “baseline scan.” After registration, the three combined masks (e.g., Figs. 2B1–3) were merged into a single “integrated mask” (e.g., Fig. 2E2). The integrated mask for the eye was then applied onto each registered CCFD binary map to exclude all potential problem areas and ensure that only areas with meaningful CC measurements across all scans were compared in later steps.

**Figure 3.**
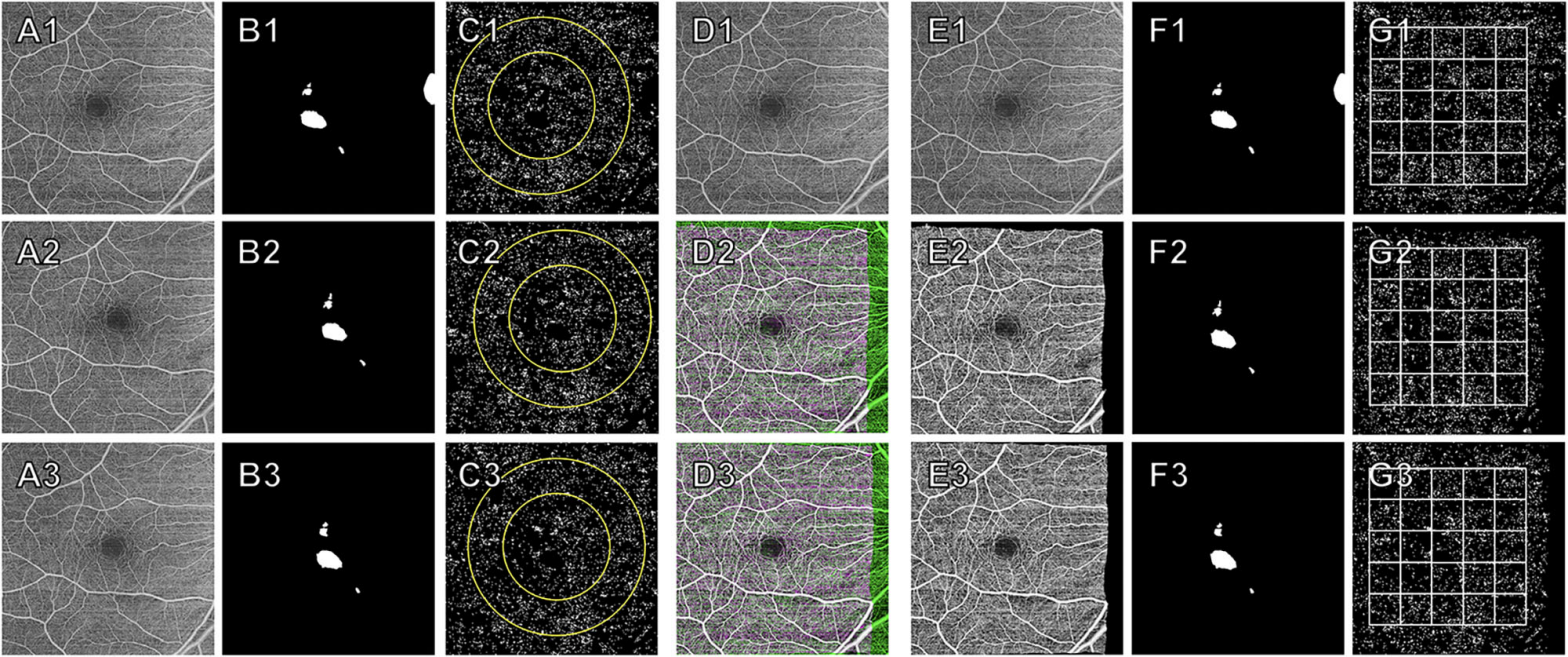
Workflow for registering three poorly-aligned repeated scans followed by the use of an integrated mask and a grid to quantitate choriocapillaris flow deficits (CCFDs). The scan was found to contain persistent hypertransmission defects (hyperTDs), hyperreflective foci (HRF), and calcified drusen (CaD). (**A1**–**3**) *En face* retinal vasculature images of the three sequential scans. (**B1**–**3**) Combined masks of HRF, hyperTDs, CaD, and peripapillary atrophy (present only in **C1**) for each scan. (**C1**–**3**) CCFD binary maps, with 3 mm and 5 mm fovea-centered circles, of each scan after applying their respective combined masks (**B1**–**3**). (**D1**–**3**) Registration of *en face* retinal vasculature images of the three repeat scans. (**E1**–**3**) *En face* retinal vasculature images showing only the overlapping regions after registration was completed. (**F1**–**3**) Registered combined masks for each scan. (**G1**–**3**) Registered CCFD binary maps after applying the case's integrated mask (merged image of the registered combined masks; not shown) with this eye's fovea-centered grid.

To create the grid, a target box, measuring 74 × 74 pixels (about 0.9 × 0.9 mm) in size (center black box in Fig. 2E1), was centered at the fovea. Then, boxes of equal size were propagated outward to the edges of the registered scan region (Fig. 2E1). The size of the box (74 × 74 pixels) was empirically chosen, balancing the need for a sufficiently localized region and a sufficiently representative one. Our choice also ensured that a set of 25 boxes would fit in the overlap of the registered scans, even if the individual scans were not perfectly centered (e.g., Fig. 3D2–3). The dimension of a grid consisted of 5 × 5 boxes, which is about 4.5 × 4.5 mm. Once the grid was created for each eye, it was overlaid onto the registered, fully masked CCFD binary maps (Fig. 2F1–3) to obtain CCFD% measurements within each box and for the total grid area for each scan.

For statistical analyses, means and standard deviations of the CCFD% measurements, along with their intraclass correlation coefficients (ICCs), were calculated for each region of interest (ROI). ICC was defined as the ratio of the total variance less the residual variance (i.e., the variance due to the repeated scans) to the total variance,^59^ and parametric bootstrapping based on 100,000 bootstrap samples was used to obtain 95% confidence intervals for each ICC. The different ROIs included the 3-mm and 5-mm fovea-centered circles, any single box of the grid, and the total grid area. For assessing any differences in CCFD% measurements between the three groups of eyes for each ROI, the mean differences were estimated using linear mixed model (LMM) analyses with random intercepts for patients, eyes clustered within patients, and grid boxes clustered within eyes (as applicable). Tukey's method was used to adjust for multiple comparisons when comparing estimated mean CCFD% among the three groups of eyes for each ROI. Finally, to understand the applicability of this grid method to longitudinal studies, the MDC_95_ was obtained for each ROI using LMMs (unconditional/variance components models) and defined as √2 × 1.96 × residual standard deviation (i.e., the standard deviation attributable to repeated scans of the same eye at the same visit under the same conditions).^60^

### Validation With Drusen-Resolving Cases

The purpose here is to demonstrate the validity of the proposed grid-based method for analyzing CCFD% longitudinally using a set of eight previously published cases in which drusen resolved without any anatomic sequelae.^53^ The scans from before and after the resolution of drusen were analyzed as described above except that only one scan was analyzed for each baseline and follow-up visit. For each eye, the target box was centered on a druse that was present at the baseline visit but resolved at the follow-up visit. Then, boxes of equal size were propagated outward to build a grid over the registered scan area without an incomplete box being positioned at the edge of the grid.

For statistical analyses, LMMs and the above-derived MDC_95_ values were used to compare changes between visits in CCFD% measurements from the target boxes with CCFD% changes in the surrounding boxes, which served as controls. Results were obtained with and without the use of the compensation strategy for OCT signal attenuation as previously described.^53^^,^^55^ Statistical analyses were performed using R version 4.4.1 with the lme4, broom.mixed, and tidyverse packages.^61^^–^^64^ A two-sided *P* < 0.05 was considered statistically significant.

## Results

Ninety eyes of 63 patients (30 normal eyes, 30 eyes with iAMD, and 30 eyes with hyperTDs) were used to evaluate the variability of the CCFD% measurements. The mean (SD) axial length was 23.75 (1.19) mm (range, 21.39–25.95 mm). The mean (SD) age of the enrolled patients was 74.8 (8.59) years, with 61% being women. Among three groups, the mean (SD) ages were 66.1 (6.1), 76.5 (6.2), and 79.7 (6.1) years in groups of normal, iAMD, and hyperTDs eyes, with 57%, 73%, and 63% being women, respectively. Within the 3-mm circle, mean (SD) transformed drusen sqrt area and cubrt volume measurements of the iAMD group were 1.29 (0.49) mm and 0.43 (0.15) mm, respectively. For the hyperTD group, the mean (SD) transformed drusen area and volume measurements were 0.83 (0.63) mm and 0.30 (0.20) mm, respectively. Within the 5-mm circle, the mean (SD) transformed drusen area and volume measurements of the iAMD group were 1.47 (0.56) mm and 0.47 (0.15) mm, respectively. For the hyperTD group, the mean (SD) transformed drusen area and volume measurements were 1.18 (0.72) mm and 0.38 (0.20) mm. The mean (SD) sqrt hyperTD area in this group was 1.15 (0.79) mm.

### Reliability Assessment

Figures 2 and 3 depict two examples of eyes with hyperTDs with three repeated scans showing the overall workflow of creating masks, registering scans, and applying each case's grid.

Table 1 shows the means and variability results (SDs, ICC, and MDC_95_) for the 90 eyes. The average CCFD% measurements were very similar between each ROI for the normal eyes alone and for all eyes taken together. The reliability of the CCFD% measurements for all sets of eyes and ROIs was very high (all ICC values ≥0.81), with most ICC values being ≥0.90. Notably, even though iAMD and hyperTD eyes showed greater variability in CCFD% than normal eyes, a greater proportion of the variance for the normal eyes was attributable to the residual variance (i.e., the variance due to repeated scans) than either iAMD or hyperTD eyes. This explains the lower ICC values alongside the lower SDs for the normal eyes for each ROI. Nonetheless, the ICC and MDC_95_ values were highly correlated and inversely related across the sets of eyes and ROIs. Of note, the single grid box ROI, which measured the smallest area, had the greatest CCFD% variability (lowest ICC values; highest MDC_95_ values) for each set of eyes, while the 5-mm circle and the total grid area had the lowest variability and were very close to each other for all sets of eyes. The latter finding was expected given that the area of grid (19.71 mm^2^) was nearly the same size as the 5-mm circle (19.63 mm^2^) and supports the expectation that no significant CCFD% information is lost through the registration and grid application processes.

**Table 1.** Choriocapillaris Flow Deficit Measurements Across Eye Groups

	Normal Cases (*n* = 30 Eyes)			iAMD Cases (*n* = 30 Eyes)			HyperTD Cases (*n* = 30 Eyes)			All Cases (*n* = 90 Eyes)		
Region of Interest	Average CCFD% (SD)	MDC_95_	ICC (95% CI)	Average CCFD% (SD)	MDC_95_	ICC (95% CI)	Average CCFD% (SD)	MDC_95_	ICC (95% CI)	Average CCFD% (SD)	MDC_95_	ICC (95% CI)
3-mm circle	8.23% (1.63%)	1.96%	0.82 (0.66, 0.90)	11.67% (4.14%)	1.70%	0.98 (0.96, 0.99)	9.76% (2.52%)	1.89%	0.93 (0.86, 0.96)	9.89% (3.26%)	1.85%	0.96 (0.95, 0.98)
5-mm circle	8.06% (1.11%)	1.36%	0.81 (0.65,0.89)	9.74% (2.02%)	0.85%	0.98 (0.96, 0.99)	9.30% (1.80%)	0.97%	0.96 (0.92, 0.98)	9.03% (1.83%)	1.08%	0.96 (0.94, 0.97)
Any box	8.10% (2.74%)	3.35%	0.81 (0.78, 0.83)	9.91% (4.62%)	3.63%	0.92 (0.91, 0.93)	10.92% (5.44%)	4.67%	0.90 (0.89, 0.92)	9.65% (4.57%)	3.93%	0.90 (0.89, 0.91)
Total grid	8.10% (1.17%)	1.40%	0.82 (0.66, 0.90)	9.89% (1.98%)	0.91%	0.98 (0.95, 0.99)	10.53% (2.04%)	1.21%	0.95 (0.91, 0.97)	9.51% (2.05%)	1.19%	0.96 (0.94,0.97)

Table reports the observed averages and standard deviations, ICCs with 95% parametric bootstrap CIs, and 95% MDC_95_ of CCFD% for each ROI and for each group of eyes. The table shows excellent reliability for each ROI across repeated scans as measured by ICC.

Additionally, Figures 2 and 3 show that the CCFD binary maps of the repeated scans before and after registration are qualitatively very similar. Table 2 shows the LMM analyses in which there were statistically significant differences in CCFD% between the normal and iAMD eyes (estimated mean difference ≥1.58% and *P* ≤ 0.006) and between the normal and hyperTD eyes (estimated mean difference ≥1.42% and *P* ≤ 0.036) for all ROI, but no significant differences between the iAMD and hyperTD groups in any ROI (*P* values ranged from 0.41 to 0.89).

**Table 2.** Comparison of Choriocapillaris Flow Deficit Measurements Between Eye Groups

Region of Interest	iAMD – Normal (95% CI)	*P* Value	HyperTD – Normal (95% CI)	*P* Value	HyperTD – iAMD (95% CI)	*P* Value
3-mm circle	2.96%	0.005	2.30%	0.036	−0.65%	0.57
	(0.78% to 5.13%)		(0.13% to 4.48%)		(−2.21% to 0.90%)	
5-mm circle	1.58%	0.006	1.42%	0.015	−0.16%	0.89
	(0.40% to 2.77%)		(0.23% to 2.61%)		(−0.99% to 0.67%)	
Any box	2.02%	0.002	2.53%	<0.001	0.51%	0.41
	(0.67% to 3.38%)		(1.18% to 3.88%)		(−0.45% to 1.46%)	
Total Grid	1.92%	0.001	2.28%	<0.001	0.36%	0.59
	(0.69% to 3.16%)		(1.04% to 3.52%)		(−0.51% to 1.23%)	

Table reports the estimated mean differences and associated CIs between each group of eyes from linear mixed models with random intercepts for patients, eyes clustered within patients, and grid boxes clustered within eyes (as applicable). Tukey's method was used to adjust the CIs and *P* values for multiple comparisons. The table shows significantly greater CCFD% in eyes with hyperTDs and iAMD compared to normal eyes for all ROIs but no significant difference in CCFD% between the iAMD and hyperTD groups for any ROI.

### Validation With Drusen-Resolving Cases

Regarding the eight cases with resolving drusen, the patients had a mean (SD) age of 67.9 (2.5) years, and 62.5% were women. The mean (SD) follow-up interval was 7.99 (4.15) months, with a range of 2.76 to 15.88 months. For the uncompensated scans, the estimated mean (95% confidence interval [CI]) CCFD% in the boxes of resolving drusen (target boxes) was 34.70% (30.97% to 38.43%) at baseline and 12.20% (8.47% to 15.93%) at follow-up, while the mean (95% CI) of the other grid boxes (control boxes) was 6.90% (6.00% to 7.81%) at baseline and 8.56% (7.65% to 9.47%) at follow-up. In contrast, for the compensated scans, the mean (95% CI) CCFD% in the target boxes was 8.57% (4.84% to 12.30%) at baseline and 8.16% (4.43% to 11.90%) at follow-up, while the mean (95% CI) of the control boxes was 7.69% (6.78% to 8.59%) at baseline and 8.03% (7.12% to 8.93%) at follow-up. For the uncompensated scan pairs, there was a significant drop in CCFD% in the target boxes (mean difference: −22.50% [95% CI, −26.51% to −18.50%]; *P* < 0.001) and a slight increase in the control boxes (mean difference: 1.65% [0.74% to 2.57%]; *P* < 0.001). In contrast, for the compensated scan pairs, there was no significant change in CCFD% in the target boxes (*P* = 0.84) or in the control boxes (*P* = 0.47) between the baseline and follow-up visits.

**Figure 4.**
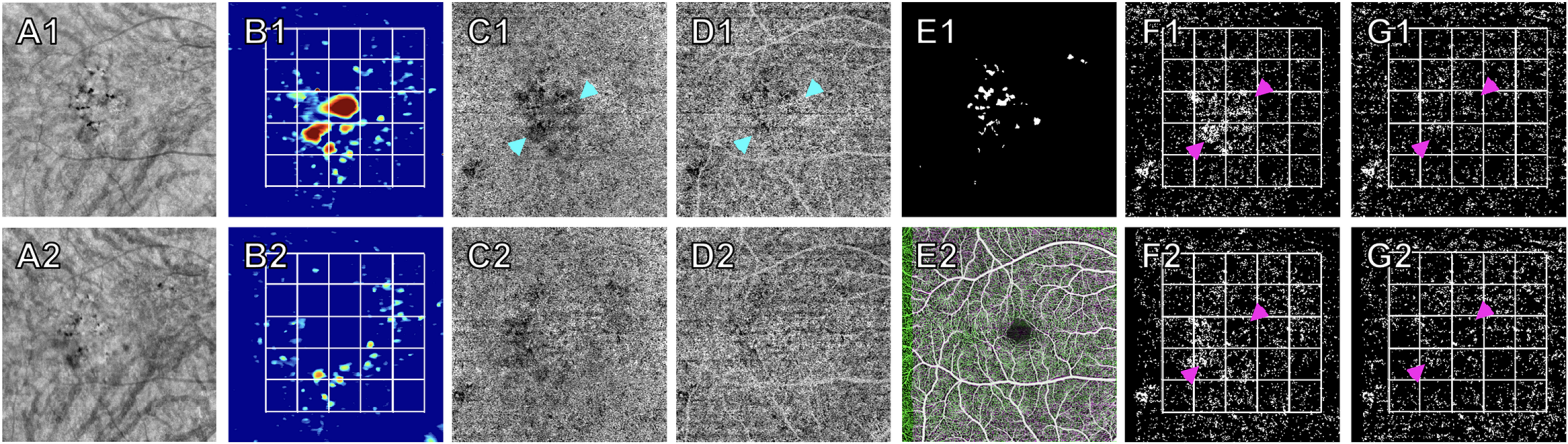
Quantitation of CCFDs within a grid before and after drusen resolution. (**A1**–**2**) En face images of the sub-RPE slabs of the baseline (**A1**) and follow-up (**A2**) visit used to create this case's integrated mask (**E1**). (**B1**–**2**) Drusen volume maps of the two visits with a grid overlay showing how the two largest drusen are included within their own boxes. (**C1**–**2**) En face images of the uncompensated CC flow slab of the two visits. (**D1**–**2**) En face images of the CC flow slabs of the two visits after compensation. The *blue arrowheads* indicate the area of prominent drusen shadowing (OCT signal attenuation) that is present in C1 and reduced in D1 after OCT signal compensation was applied using the CC structure image (not shown). (**E1**) Integrated mask created by registering and merging the combined masks (not shown) of HRF and CaD from the individual scans. (**E2**) Registration of en face retinal vasculature images of the two scans. (**F1**–**2**, **G1**–**2**) Registered, uncompensated (**F1**–**2**) and compensated (**G1**–**2**) CCFD binary maps of the two scans after the integrated mask and grid overlay were applied. *Magenta arrowheads* show dense areas of CCFDs under areas of drusen (**F1**), which decrease once the drusen resolve (**F2**) or are rendered insignificant in both visits once OCT signal compensation is applied (**G1**–**2**). Without compensation, the CCFD% in the two boxes with significant drusen at baseline were 25.60% and 29.76% while CCFD% at follow-up were 10.62% and 21.16%, respectively. After compensation, the CCFD% of these two boxes were 9.95% and 9.38% at baseline and 9.02% and 8.90% at follow-up, respectively. With compensation, the change in the individual drusen boxes was within the MDC_95_ value of 3.93%.

**Figure 5.**
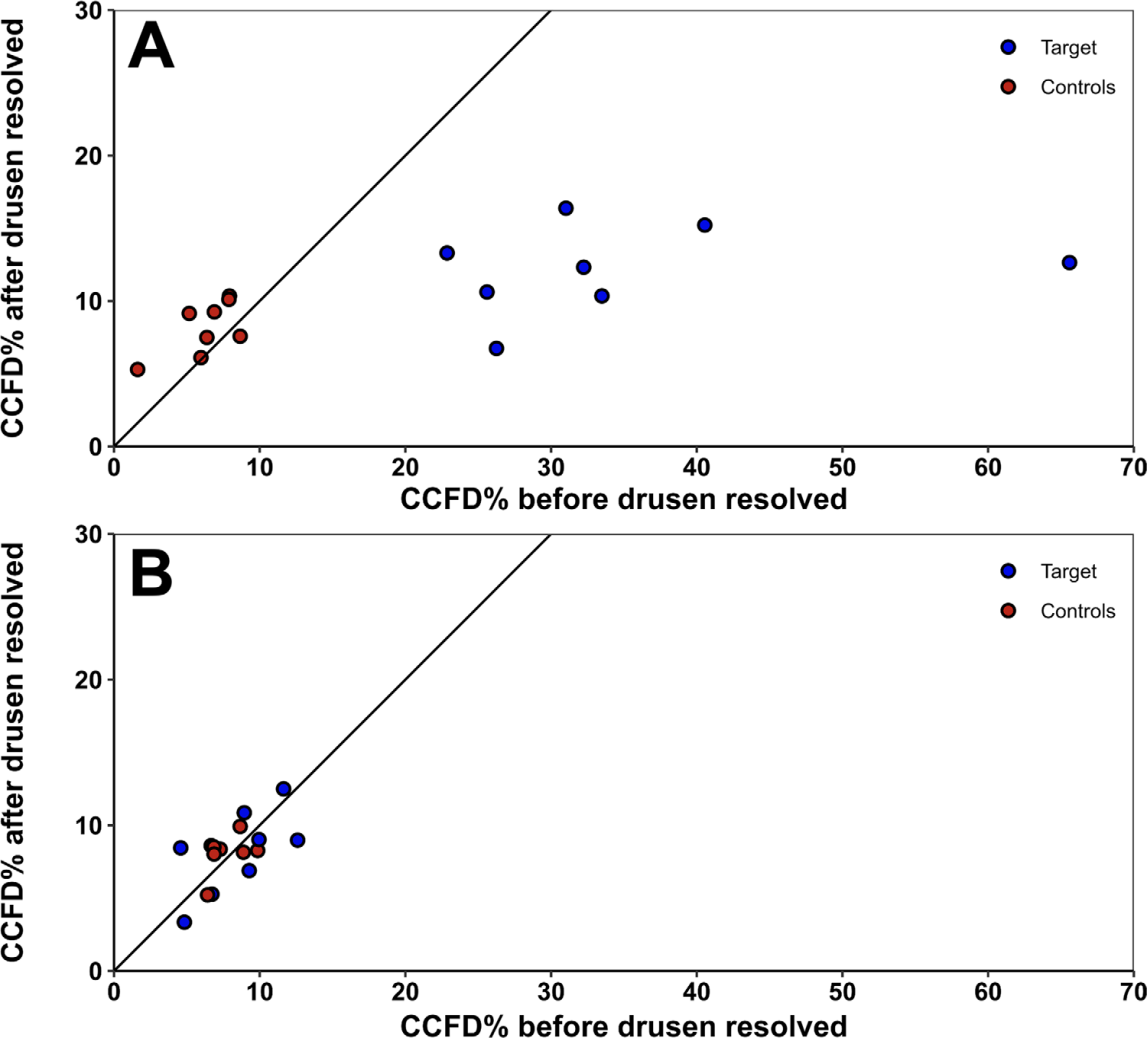
CCFD measurements before and after drusen resolved using the grid strategy. The resolving druse was contained within a target grid box, and the remaining boxes in the grid without significant drusen changes served as internal controls. Changes in CCFD% were compared in boxes where drusen resolved (target boxes), both with and without compensation. (**A**) CCFD% using uncompensated CC flow images before and after drusen resolution. The target boxes had significantly greater mean CCFD% at baseline than after the drusen resolved (*P* < 0.001) while the control boxes had a slightly higher mean CCFD% after the drusen resolved than at baseline (*P* < 0.001). (**B**) CCFD% using compensated CC flow images before and after drusen resolution. No significant differences in CCFD% were found in either the target boxes or the averages of the control boxes.

Figure 4 shows one of the eight drusen-resolving cases in which a visible decrease in drusen shadowing and an increase in CC perfusion can be appreciated on the CC flow image after compensation. Figure 5 demonstrates the change in CCFD% of the target and control boxes between visits for each eye when scans are compensated. For the uncompensated scan pairs, the target boxes show a significant drop in CCFD% measurements between visits, while the control boxes are clustered around the line of unity. When compensation is applied, both the target boxes and the control boxes are clustered at the line of unity, with no significant change between visits. When utilizing the MDC_95_ values derived above, all the target boxes of the uncompensated pairs showed CCFD% changes greater than 3.93% in magnitude (range: −9.56% to −52.95%). In contrast, none of the target boxes of the eight compensated pairs showed CCFD% changes greater than 3.93% in magnitude. When all the grid boxes were analyzed with compensation before and after drusen resolution, no significant differences in CCFD% measurements were observed (*P* = 0.51).

## Discussion

AMD progression is characterized by focal macular anatomic changes in soft drusen, CaD, HRF, BLamDs, outer retina thickness, SDDs, hyperTDs, and the onset of MNV and exudation.^3^^–^^7^^,^^9^^,^^11^^,^^12^^,^^14^^,^^16^^,^^18^^,^^48^^,^^51^ Some of these macular anatomic changes have been reported to be associated with underlying changes in CC perfusion.^12^^,^^17^^,^^38^^,^^39^^,^^43^^,^^44^^,^^46^^,^^65^^–^^67^ While different grid-based methods for the analysis of spatial localization have been reported in glaucoma^68^ and iAMD with RPD,^69^ the temporal and regional relationships between CC perfusion loss and other focal macular anatomic biomarkers have not been evaluated in longitudinal studies. As an initial step in this direction, this study introduces a novel grid-based method of registering and dividing macular scans into equivalent boxes to allow for the independent analysis of localized regions over time. We validated its use with eight previously published cases of drusen that resolve with time.^53^

This novel grid approach incorporated the ability to (1) mask out lesions that confound the accurate assessment of CCFDs, (2) compensate for OCT signal attenuation, (3) threshold and binarize scans to identify areas of CCFDs, (4) register scans so that the same macular regions are being compared, (5) create integrated masks to ensure that comparisons are only performed between shared regions, and (6) divide the scans in such a way that comparisons of target regions are in focus while regions outside the target can serve as internal controls. In developing the algorithm, we decided that registration should be performed after the calculation of CCFDs to make the workflow more efficient, as otherwise, the CCFD algorithm would need to be repeated for every registered pair of scans. With our current grid workflow, the masking, compensation, and binarization are performed on each scan separately, and then the scans are registered and an integrated mask for the set of scans to be compared is applied. Thus, whenever new comparisons need to be made, the scans’ original binary maps and per-visit masks can be registered and used repeatedly without needing to reprocess the scans to determine the CCFD results. In addition, while all the cases in this study only used grids containing 25 boxes, it is worth noting that an individual box size of 74 × 74 pixels can create a grid of up to 36 boxes in a 6-mm × 6-mm field of view, depending on the extent of overlap between registered scans and the relative location of the target box in the scan.

To understand the reproducibility and reliability of our grid approach, we utilized three same-day repeated scans of 90 eyes with varying levels of pathology, ranging from normal eyes to eyes with different degrees of HRF, CaD, and hyperTDs. Our grid workflow was shown to have good to excellent reliability for all ROIs across all groups of eyes (all ICC values ≥0.80). The high degree of reliability gives us confidence that this approach is a useful way of studying the association between localized macular anatomic changes with CCFDs over time. An important benefit of our grid method in longitudinal studies is related to the MDC_95_ values determined for each ROI. The MDC_95_ values (Table 1) provide a reference point to determine when a “true” change (one that is outside of measurement variability) has occurred between visits. In line with this, we have shown that a minimum difference of 3.93% in CCFD% within a given box would need to be exceeded before the change in CCFD% could be considered significant with a 95% confidence. Similarly, a minimum difference of 1.19% needs to be seen for the total grid area, 1.85% for a 3-mm circle, and 1.08% for a 5-mm circle for changes between scans to be deemed significant.

As a proof of concept and a way to validate the utility of our approach, we applied the grid workflow to eight previously published cases of resolving drusen to determine whether the results aligned with the published findings that CCFD% under drusen are artifactually increased due to the shadowing effects of drusen, and a compensation strategy was able to correct for this attenuation.^53^ As expected, without OCT signal compensation, LMMs showed there was a significant drop in CCFD% in the target boxes between visits, but once compensation was applied, the target boxes showed no significant difference between visits. We obtained similar results in terms of our MDC_95_ values. These results are in contrast to the findings by Nassisi et al.^39^ and Tiosano et al.,^70^ who showed there was a unique increase in CCFD under and immediately around areas of drusen. However, unlike our study, the study by Nassisi et al.^39^ did not compensate for the shadowing effect of drusen, while both of them did not mask out areas of HRF or CaD.^70^

Of note, while it was not the primary intention of this study, we compared the CCFD% measurements between our three groups of eyes. We found that while both iAMD and hyperTD eyes had greater CCFDs than normal eyes in all ROIs, there were no significant differences between iAMD and hyperTD eyes in any ROI (Table 2). While the first finding directly aligns with previous studies that have shown an increase in both local and global CCFD% in iAMD eyes and in those with atrophy compared with normal eyes, the latter finding presents an interesting contrast with previously published studies that have shown that increased CCFD% are related to atrophy growth.^33^^,^^38^^,^^40^^,^^41^^,^^43^^–^^46^ This may be explained in a number of ways. For example, most studies do not mask out HRF and CaD, even though their high optical scattering properties may interfere with the accurate quantification of CCFDs.^38^^,^^42^^,^^44^^,^^46^ That is, HRF and CaD cannot be adequately compensated using the currently available compensation strategies and may lead to artifactually increased measurements of CCFDs under those lesions.^5^^,^^7^^,^^71^ This is significant because there is an increased prevalence of HRF and CaD as eyes progress to late AMD,^3^ thus allowing for the possibility of increased CCFD% measurements in eyes with hyperTDs compared to eyes with iAMD, as seen in other studies. However, the most likely explanation for the lack of global differences in CCFD% between eyes with iAMD and those with persistent hyperTDs is that our measurements were performed without targeting specific regions, such as hyperTDs, within the scan that contains the specific anatomic changes that distinguish iAMD from eyes with persistent hyperTDs. Future studies will focus on these specific anatomic changes and determine the temporal relationship between CCFD% measurements and the onset and progression of these anatomic changes.

Our study has some limitations. First, we are limited by the cases we included in the reliability portion of this study. While a strong effort was made to ensure that a wide range of cases were included, we acknowledge that our resultant ICC and MDC_95_ values may differ slightly if additional cases were included. However, we would anticipate that including more cases would likely increase the ICC values and reduce the MDC_95_ values, so if a difference is detected using the current values, then we could remain confident that a real difference is present. Second, the box size used in this study, although empirically chosen, lacks a true anatomic correlation. As such, future studies may opt to use a different box size. In such cases, new ICC and MDC_95_ values would need to be calculated for different box sizes. Third, we focused here on analyzing a single target box (i.e., lesion of interest) within each scan. However, it is not difficult to generalize the approach. For instance, a new grid could be made for each lesion of interest in the scan, with a target box appropriately centered over the lesion of interest. Finally, when using this grid strategy in different eyes, variability in the lateral scale of the images, particularly in the absence of axial length corrections,^57^^,^^58^ can result in different areas covered by the grid among eyes. Thus, when comparing the area or volume results among different eyes, readers should interpret the results with caution. However, since our study focuses on CCFD%, which is a density measurement, axial length variations should have a minimal effect.^72^ Moreover, this limitation should not significantly affect the conclusion when studying longitudinal changes in the same eye.

Overall, by establishing the reliability of CCFD% measurements from our grid workflow strategy, determining reference values (MDC_95_ values) that tell us if a local CCFD% change between visits could be considered significant, and validating the grid workflow with previously published cases of drusen resolving over time, we have demonstrated that our grid approach can be used longitudinally to study the relationship between focal changes in CCFD% and focal anatomic changes over time. In the future, we will apply this grid approach to iAMD eyes as they progress to late AMD. Doing so will allow us to create a comprehensive temporal explanation of whether the localized changes in the CCFD% correlate with the onset or progression of the characteristic anatomic changes in AMD, including the onset and progression of RPD/SDD, BLamDs, photoreceptor loss, HRF, soft drusen, CaD, and hyperTDs. If changes in CCFD% measurements precede the anatomic changes of interest, such as the onset of persistent large choroidal hyperTDs, then we may be able to predict the onset of these changes and possibly intervene earlier to prevent their onset and progression.
